# Graves' Ophthalmopathy: A Review of Immunogenetics

**DOI:** 10.2174/138920211798120844

**Published:** 2011-12

**Authors:** Omid Khalilzadeh, Sina Noshad, Armin Rashidi, Aliakbar Amirzargar

**Affiliations:** 1 Molecular Immunology and Immunogenetics Research Center, Tehran University of Medical Sciences, Tehran, Iran; 2 Immunogenetics Lab, Department of Immunology, School of Medicine, Tehran University of Medical Sciences, Tehran, Iran; 3 Department of Internal Medicine, Eastern Virginia Medical School, Norfolk, VA, USA

**Keywords:** Cytokine, Graves, immunogenetics, ophthalmopathy, polymorphism.

## Abstract

Graves’ disease (GD) is the most common cause of thyrotoxicosis and often involves the orbits. Graves’ ophthalmopathy (GO), also known as Thyroid Eye Disease (TED), can be clinically significant and advance to sight-threatening stages. Our knowledge of the immunogenetic pathophysiology of GO is rapidly expanding. The present review is an attempt to summarize the current state of knowledge on the immunogenetics of GO. First we briefly review the epidemiology and clinical importance of GO, and then we describe in detail the macromolecular pathogenesis and finally immunogenetics of GO. Discrepancies between the results from various reports and the limitations of the available data are discussed. In particular, there is a scarcity of data from non-Asian populations. While several studies have demonstrated significant associations between polymorphisms in certain genes (especially CTLA-4, HLA-DRB-1, and TNF-α), there is a need for studies that investigate the relationship between polymorphisms and both serum and local concentrations of the resulting proteins. A complete understanding of GO susceptibility and pathogenesis has not been yet possible due to a number of important knowledge gaps that need to be filled by future research.

## 1 INTRODUCTION: GRAVES’ DISEASE

Graves’ disease (GD), the single most common cause of thyrotoxicosis, is an autoimmune disease of aberrant antibody production [1]. Antibodies directed against thyrotropin receptor (TSHR) target the endothelial surface of thyroid follicular cells where these receptors are most abundant [2]. Although almost any organ may be involved in GD, GD is classically described as a syndrome consisting of hyperthyroidism, goiter, orbitopathy, and dermopathy. Follicular hyperplasia, intracellular colloid droplets, cell scalloping, a reduction in follicular colloid, and a patchy T-cell-predominant lymphocytic infiltration characterize the histology of the thyroid gland in GD. GD is primarily a T helper-2 (Th2) autoimmune disease since antibody secretion against TSHR is the cornerstone of the disease pathophysiology [3]. 

Several risk factors have been identified for GD. Genetic susceptibility is an important concept. The disease tends to run in families (concordance rate of 20%-40% in monozygotic twins and greater than 10% in siblings) and has a predilection to women (female to male ratio of 7:1) [4-5]. Infections of the thyroid gland have been proposed to trigger the autoimmune cascade leading to GD, but the available evidence is slim [6]. Many patients with GD report a history of some type of psychological stress before the onset of the disease. Rebound immunologic hyperactivity following stress-related, corticosteroid-induced immune suppression has been proposed as a mechanism for the role of stress in GD [7-9]. Estrogen-induced immunologic reactivity has been linked to GD, consistent with the observation that GD affects women much more commonly than men. Interestingly, however, susceptibility to GD continues after menopause, suggesting that the X-chromosome rather than estrogen might be the source of enhanced susceptibility [10]. Smoking is another risk factor for GD, with largely unknown mechanisms [11-12]. 

The onset of GD in up to 30% of women is within a year after pregnancy [13]. Although the mechanisms for this interesting association are not clear, the presence of fetal cells in maternal tissues after pregnancy (fetal microchimerism) might play a role [14]. Finally, iodine and iodine-containing drugs such as amiodarone may predispose a susceptible individual to GD [15]. It has been suggested that iodine (and amiodarone) damages thyrocytes and makes thyroid antigens exposed to the immune system [16]. In the iodine-deficient susceptible patient, iodine can precipitate GD by allowing TSHR antibodies to more effectively stimulate thyroid hormone formation. 

The literature on GD is extensive. The focus of our attention in this article is on a specific complication of GD, namely Graves’ ophthalmopathy (GO) or Thyroid Eye Disease (TED). First we briefly review the epidemiology and clinical importance of GO, and then we will describe in detail the macromolecular pathogenesis and finally immunogenetics of GO. 

## 2 GRAVES’ OPHTHALMOPATHY: EPIDEMIOLOGY AND CLINICAL IMPORTANCE

The GD process is not limited to the thyroid gland. In a subset of patients GD also involves the pretibial skin and the orbit, the latter known as GO or TED [17]. Orbital involvement is not merely a byproduct of hyperthyroidism; rather, the same underlying immune processes involving TSHR antibodies are active in the orbits [17]. TSHR antibody titers seem to be positively correlated with clinical features of GO, whereas thyroid stimulating immunoglobulin (TSI) and thyroid peroxidase (TPO) antibody do not [18].

The degree of eye involvement among GD patients varies considerably from very mild cases with tenuous changes only detected on imaging, to more severe sight-threatening forms with optic nerve compression [17]. Clinically evident GO can be detected in 25-50% of patients sometime in the course of disease but only 3-5% experience debilitating forms [1]. In the presence of lid retraction, lid lag, proptosis, extraocular muscle involvement, or optic nerve dysfunction, a clinical diagnosis of GO can be made [19]. In a retrospective cohort of 120 subjects with GO, the most common presentation was lid retraction (90%), followed by proptosis (62%) [20]. These manifestations are due to mechanical compression of the orbit stemming from expansive changes in orbital connective tissues and extraocular muscles. With increased orbital pressure, lymphatic and venous return is hindered and congestive swelling develops [21]. Patients often complain of redness, swelling, grittiness, foreign body sensation, dryness, excessive tearing, restricted eye movements, diplopia, and inability to close the eyes during sleep [17]. Of the extraocular muscles involved, inferior rectus is the most commonly affected muscle followed by medial rectus, superior rectus, and lateral rectus, respectively [22].

A number of scoring systems has been developed to define the severity of GO, among which the NOSPECS and Clinical Activity Score (CAS) systems have been widely used in research and practice. The NOSPECS system (1977) uses a 7 class scheme from 0 (no signs or symptoms) to 6 (sight loss) [23]. Classes 2 through 6 are further divided by grades a, b, or c; for example in class 5 (corneal involvement), grades a, b, and c correspond to “stippling of cornea”, “ulceration”, and “clouding, necrosis, perforation”, respectively [23]. The CAS system (1989), on the other hand, adopts a ten-item questionnaire with the sum of all positive scores being indicative of disease severity [24]. The 10 items of CAS concern the following four signs of inflammation: pain (2 items), redness (2 items), swelling (4 items), and impaired function (1 item for movement restriction, and 1 item for impaired visual acuity) [24-25]. 

Similar to other autoimmune conditions, GO has a multifactorial etiology; genetic and environmental factors interact in concert and trigger a deviant process that presents self-antigens as non-self [26]. Risk factors for GO development or progression include ethnicity (African-American > European > Asian), sex, treatment with radioactive iodine, and hyper- or hypothyroidism [27]. Certain anatomical features of the orbital walls (i.e. wide angle of the lateral wall) have also been linked to more severe forms since orbits with these features cannot provide adequate space for orbital expansion [27]. Among modifiable risk factors, smoking has been repeatedly linked to both development and progression of GO with odds ratios ranging from 7.7 to 20.2 [11, 28]. Smoking cessation has been consistently advised at primary, secondary, and tertiary levels of prevention [29]. Like other diseases of autoimmune origin, women are more likely to develop GO [1, 27]. An annual age-adjusted incidence of 2.9 per 100,000 in men and 16 per 100,000 in women has been reported for GO [30]. Compared with men, the peak incidence of GO is about five years earlier in women than in men [30]. With further follow-up of a cohort of patients with GO in Olmsted for 9.8 years, GO regressed in most cases, however at least one third of patients were discontented about the appearance of their orbit [31]. Serious complications including permanent sight loss (2.2%), and permanent diplopia (2.2%) were also noted, albeit rarely [31]. Although women are more affected, men are more likely to develop severe GO [27, 32]. In a cohort of 2,045 subjects with GD, the rate of GO with NOSPECS classes 4-6 (severe disease), was 30.4% and 21.3% in men and women, respectively [32]. Finally, radioactive iodine treatment of hyperthyroidism has been linked to the progression and worsening of GO. Radioiodine therapy seems to induce substantial increases in the serum concentrations of TSHR antibodies, which might trigger or exacerbate GO [33]. Risk factors that increase the likelihood of this complication include smoking, moderate to severe GO [34], and a high baseline serum concentration of triiodothyronine [35].

## 3 GRAVES’ OPHTHALMOPATHY: MACROMOLECULAR PATHOGENESIS

The TSHR-major histocompatibility complex class II (TSHR-MHC II) complex is internalized and processed by antigen-presenting cells (APC) and presented primarily to CD4+ T-cells *via *the T-cell receptor (TCR). Activated T-cells recruit B-cells through CD40-CD154 ligation. B-cells differentiate into plasma cells *via *interferon γ (INF- γ) and interleukin 2 (IL-2), activated T-cells recognize THSR on the orbital fibroblasts, and hence a site-specific immune response is initiated [17]. Little is known about the details of the events leading to the formation of TSHR-MHC II anomalous connection, nonetheless a number of mechanisms have been proposed. Infections can (*i*) cause an imbalance in cytokine production, (*ii*) induce non-specific activation of T- and B-cells through superantigens and mitogens, and (*iii*) alter self-antigens to a degree that are perceived as non-self [1]. Some studies have found a link between a history of infestation with *Yersinia* species and GD [6, 36]. *Yersinia entrocolitica *displays membrane proteins similar to TSHR (a requirement for the process of molecular mimicry) and can bind to TSH, [37].

A compelling body of evidence points toward a central role for orbital fibroblasts in the pathogenesis of GO [1]. Higher levels of TSH mRNA have been observed in GO fibroblasts compared with their normal counterparts [38]. They also express higher levels of insulin-like growth factor-1 receptor (IGF-1R) that may be recognized as an autoantigen and contribute to GO pathogenesis [39] (see below). There are two subpopulations of fibroblasts with distinct behaviors in response to inflammatory cytokines: The Thy-1+ (CD90) subpopulation produces high levels of hyaluronan, whereas the Thy-1- cells are capable of differentiating into adipose tissue [40]. Hyaluronan, a hydrophilic glycosaminoglycan, absorbs high quantities of water and contributes to the expansive remodeling in the orbital space and within muscle fibers [41]. Hyaluronan production is enhanced with IL-2 and transforming growth factor β (TGF-β) secretion [42]. The process of adipogenesis, on the other hand, is mediated through activation of peroxisome proliferator-activated receptor gamma (PPAR- γ) [43]. PPAR- γ not only drives adipogenesis, but has immunomodulatory roles as well. When activated, PPAR- γ abates chemokines CXCL9, 10, 11, and INF- γ that are characteristic of T helper-1 type (Th1) response, hence contributing to the transition from Th1- to Th2-mediated immunity [44]. While IL-6 enhances adipogenesis [45], tumor necrosis factor α (TNF-α), TGF-β, and INF- γ inhibit the process [46]. Mature adipocytes augment the expression of TSHR, and significantly contribute to the maintenance of the disease process [47].

Recruitment and infiltration of assorted classes of immune cells in the orbit is dependent upon an abundant pool of the produced cytokines. Cytokines have important putative roles in GO development, progression, and regression and are produced by activated T-cells (mostly CD4+ cells), macrophages, B-cells, and orbital fibroblasts [48-50]. Th1 lymphocytes and their associated family of cytokines [TNF-α, interleukin 1β (IL-1β), INF- γ, IL-2, and interleukin 12 (IL-12)] [51] predominate the orbit early in the course of the disease and promote the active phase of GO. Later in the course of the disease, the Th2 family [with interleukin 4 (IL-4), interleukin 5 (IL-5), and interleukin 10 (IL-10)] [52] takes over, a transition associated with maintenance and resolution of the disease [53-54]. The orbital fibroblasts directly interact with activated T-cells through CD40-CD154 ligation. Indeed, the orbital fibroblast of patients with GO show higher levels of expression of CD40 than their normal counterparts [55]. CD40 belongs to the TNF-R family of receptors and engages in B-cell regulation, proliferation, antibody production, and conversion to memory cells [26]. With ligation of surface CD40 with CD154, the incited fibroblasts produce interleukin 6 (IL-6), interleukin 8 (IL-8), and macrophage chemoattractant protein-1 (MCP-1) [55].

As mentioned above, TSHR is the dominant autoantigen responsible for the brisk immune activation in GO. However, there seem to be other self-antigens involved in the disease process. When IGF-1R (expressed on the surface of fibroblasts) is activated by antibodies extracted from sera of GD patients, T-cell chemo-attractants are upregulated [56]. Interestingly, TSHR and IGF-1R co-localize on the outer and inner membranes of both thyrocytes and orbital fibroblasts, raising the possibility of functional interactions between the two receptors [57]. Antibodies directed against extraocular muscle proteins (namely G2s and Fp) [58] have also been linked to GO. There have been arguments, however, that in the presence of an intact cell structure, a humoral response against intracellular proteins such as G2s and Fp is unlikely. A plausible explanation is that these proteins are released in the serum from damaged cells late in the disease process and are more indicative of disease severity than its causal underpinnings [21, 58]. 

What makes the connective tissue of the eyes particularly susceptible to GD? Recent studies have shed light on this puzzling question. Orbital fibroblasts manifest increased production of prostaglandin E2 when exposed to IL-1β or leukoregulin in-vitro [48]. These fibroblasts exhibit a more vigorous inflammatory response than those from the skin or lungs when stimulated by platelet-derived growth factor-BB [44]. Collectively, orbital fibroblasts are unique in the sense that they not only manifest an intense inflammatory response, but are also actively involved in the process by secreting various cytokines and chemoattractants [39].

## 4 GRAVES’ OPHTHALMOPATHY: IMMUNOGENETICS

In this section, we provide a detailed review of the immunogenetic associates of GO. Genetic factors related to cytokines, human leucocyte antigens (HLA), cytotoxic T-lymphocyte antigen 4 (CTLA-4), and other loci [Toll-like receptors (TLR), B7 molecules, etc] are discussed in separate sections. A summary of the relevant studies is provided in Table **1**. Fig. (**1**) provides a schematic summary of the most important immunogenetic mechanisms in GO susceptibility and progression. 

### 4.1 Cytokines

Considering that GO is an autoimmune condition, any imbalance between the production of pro- and anti-inflammatory cytokines can alter the downstream cascade of reactions and trigger the autoimmune response [59-60]. Therefore, single-nucleotide polymorphisms in the cytokine genes which influence the expression of the pro- and anti-inflammatory cytokines can protect from or promote the development of GO [59]. In an exhaustive immunogenetic series of studies in Iran, we have explored the association between cytokine gene polymorphisms and GO [61-63]. We identified significant associations between a number of polymorphisms in pro- and anti-inflammatory cytokine genes and GO (Table **1**). 

Cytokines released predominantly by leukocytes infiltrating the retro-orbital tissues are likely to play a key role in the cascade of the autoimmune reactions in the orbit [64]. Although several significant associations between genetic polymorphisms in cytokine genes and GO have been described, the immediate consequences of cytokine gene polymorphisms are not well studied. Such consequences include changes in serum and local concentrations of the cytokines as well their activity levels. The available literature on this subject is scarce, and a comprehensive study would be of great value. Overall, there are many contradictory results regarding the association between polymorphisms of cytokine genes and GO, likely reflecting different genetic patterns of susceptibility among different ethnic groups [65-66].

Among several cytokines studied, the role of polymorphisms in the cytokines of the IL-1 family and TNF-α in genetic susceptibility to GO seems to be the strongest. The TNF gene codes for TNF-α, a key proinflammatory cytokine that has also been the target of a new therapy for GO [67]. A group of authors from Japan showed a positive association between two polymorphisms in the 5′-promoter/enhancer region (-1031T/C and -863C/A) of the TNF gene and the development and severity of GO [68]. Their results at position -863C/A were replicated by a study from China [69]. However, another study in Polish-Caucasian patients did not show a significant association at this position [70]. In the latter study, a significant association was found only for the polymorphism at position -238G/A [70]. 

In a study from Japan, eight different alleles named as IFN-γ*1 to IFN-γ*8 were identified, among which IFN-γ*3 and IFN-γ*5 were associated with GO [71]. A study from Iran showed a significant association between GO and a polymorphism at IFN- γ UTR 5644A/T [63]. 

The major members of the IL-1 superfamily are IL-1α, IL-1β, and the IL-1 receptor antagonist (IL-1RA). IL-1α and -β are pro-inflammatory cytokines and the IL-1RA competes for receptor binding with IL-1α and IL-1β [72]. There are significant differences in the expression and control of the IL-1RA gene between orbital fibroblasts derived from patients with active GO and healthy individuals [73]. Cultured retro-orbital fibroblasts derived from patients with GO express and release significantly lower quantities of intracellular and soluble IL-1RA compared with normal fibroblasts [74-75]. It is suggested that an imbalance between IL-1 and IL-1RA may play an important role in the pathogenesis of GO [73]. Although IL1-RA gene polymorphisms have not been linked with GO in all studies [76-78], a significant association was found in a study from Iran [61]. IL-1 stimulates retro-orbital fibroblast proliferation, glycosaminoglycan synthesis, and expression of immunomodulatory molecules by retro-orbital fibroblasts [42, 79]. IL-1 can induce the expression of adhesion molecules, cytokines, complement regulatory proteins, and stress proteins in thyroid cells and retro-orbital fibroblasts [80]. Polymorphisms in IL-1-α and IL-1-β genes have shown conflicting associations, i.e. both negative [59, 77] and positive [61, 66, 81], with GO. A recent meta-analysis covering data from Taiwan, Iran, and Poland showed that the rs1800587 (IL1-α, -889 T/C) and rs16944 (IL-1β, -511 A/G) polymorphisms may confer susceptibility to GO in the Asian population [66]. 

A summary of other cytokines with significant associations with GO is provided in Table **1**.

### 4.2 CTLA-4

The CTLA-4 gene encodes for a negative regulator of the T-cell immune response and seems to be an immunogenetic associate of several autoimmune diseases [82-84]. The A/G polymorphism at position 49 in exon 1 has been the focus of many studies [85-87]. This polymorphism results in an amino acid exchange (Threonine/Alanine) which may lead to decreased expression of CTLA-4. In a study from the UK, the 49A/G polymorphism was associated with an increased risk of GO [88]. This result was later confirmed by another study from the same research group [87] and a recent study by our group from Iran [85]. Also, the CTLA-4 A49G polymorphism might predict remission rates in patients treated with antithyroid drugs [89]. The relationship between the CTLA-4 gene polymorphisms and the development of GO has been the subject of several studies in different populations. Some [85-88], but not all [90-96], of these studies are in support of a significant association. Unfortunately, most of these studies are under-powered and have used different definitions for GO or enrolled patients with different degrees of GO severity. A meta-analysis by Bednarczuk *et al. *[97] argued against a significant association between the CTLA-4 A49G polymorphism and GO. The authors emphasized that their meta-analysis had considerable limitations due to discrepancies in design and data presentation of the individual reports.

### 4.3 HLA

HLA gene polymrphisms are one of the susceptibility factors proposed for GO. There are two types of HLA molecules: class I (A, B and C) and class II (DP, DQ and DR). HLA molecules are encoded by a cluster of genes forming a complex on chromosome 6 (MHC) [98]. HLA class II plays an important role in the activation of CD4+ T lymphocytes which have a key role in the regulation of autoimmune reactions leading to GD and GO. Therefore, HLA class II genes are one of the key candidates for the development of GD and GO [99].

A major limitation in studying autoimmune associations of different HLA alleles is the strong linkage disequilibrium between HLA alleles and alleles of undefined neighboring loci which may exert the primary effect [70, 100]. Therefore, functional studies of the biological effects of different HLA alleles are needed to determine the true effects of these potential genetic associates of GO. For example, the HLA class I antigen, HLA-B8, was described to be associated with GO [101]. However, further investigation revealed that this effect was most probably due to a linkage disequilibrium between HLA-B8 and HLA-class II molecules, especially HLA-DR3 (HLA-DRB1*03 alleles) [102-103]. There are several pieces of evidence in support of the role of HLA-DRB1 in GO [92, 102, 104-107]. Contradictory results, however, exist as well [70, 90, 92, 95]. HLA-DRB1 polymorphism at position 74 is of pathophysiological relevance, because it has a central role in autoantigen presentation by HLA-DR to T cells [108]. HLA-DR7 alleles are also reported to have a role in genetic susceptibility to GO [104, 109]. Based on isolated studies, an association between HLA-DR4, HLA-DPB 2·1/8 and HLA-DRB3 alleles and GO seems likely [99, 105, 110]. A meta-analysis in 2007 did not show any significant association between GO and polymorphisms in HLA-DR3, -DR4 and -DR7 [97]. Studies from Japan suggest that HLA-A11, -B5, -DW12 and -DR14 are associated with GO, whereas HLA-DPw2 confers a protective effect [111-112]. 

### 4.4 Other Genetic Factors

GO was recently reported to be associated with polymorphisms in the gene for intracellular adhesion molecule-1 (ICAM-1), which plays a major role in leukocyte circulation and migration [113], as well as polymorphisms in genes for lymphoid protein tyrosine phosphatase (PTPN22: a negative regulator of T-cell activation) [114], nuclear factor-kappa-B (NFκB; a transcription factor activated by various intra- and extra-cellular stimuli such as cytokines) [115], glucocorticoid receptors [116], TLR-9 (TLR: a family of pattern-recognition receptors involved in eliciting innate/adaptive immune responses and chronic inflammation) [117], CD86 (B7 molecules; factors that provide a costimulatory signal for T cell activation and survival and are ligands for CD28 and CTLA-4 on T cell surface) [118], and CD103 (Integrin-alpha E) [119]. Other reports suggest that functional polymorphisms in the PTPN22, CD40, NFκB inhibitor, MAP3K7IPT and Fc receptor-like 3 molecule (FCRL-3) genes are not associated with GO [120-122]. Theoretically, the TSHR gene is a suitable candidate for genetic susceptibility to GD because it is a thyroid-specific gene, and it is well known that antibodies against the TSHR play a major role in activation of thyroid cells and hyperthyroidism [123]. However, case-control studies have not been successful in finding an association between polymorphisms in the TSHR gene and GO [124-125]. The debate is ongoing. 

## 5 CONCLUSIONS

We provide an exhaustive review of the available studies on the immunogentics of GO. Several important patterns arise from this review. First, more than half of the studies are from Asian populations. Considering that ethnicity may play an important role in the immunogenetics of autoimmune conditions, the results from one ethnicity cannot be extended to the other ethnicities in a straightforward manner. There is a scarcity of data from Caucasian populations and a complete lack of data from Africa. Studies from these ethnicities would be illuminating. Second, the three most frequently reported associations with GO are for CTLA-4, HLA-DRB-1, and TNF-α. The interplay between these three factors might be a significant determinant of GO susceptibility and pathogenesis. However, particular caution should be practiced when considering HLA genes. These genes, as described above, tend to be in linkage disequilibrium with neighboring genes, which might also be important players in GO pathogenesis. Finally, association studies like those reviewed here only serve as a start point for further studies. No causal relationships can be extracted from a mere association. One of the best ways to enrich the results of the observed associations is by functional studies that investigate the relationship between a given polymorphism and serum level of the resulting protein. For GO, however, one also needs to know the relationship between the polymorphisms of interest and local (i.e. orbital) concentrations of the resulting protein. Very little attention has been paid to this point, a fact which has limited our understanding of the disease pathogenesis. 

## Figures and Tables

**Fig. (1) A schematic diagram summarizing the immunogenetic pathophysiology of Graves’ ophthalmopathy.**
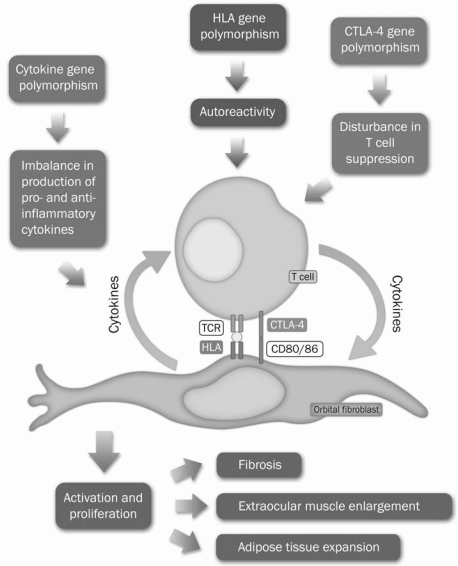
Disease manifestations are the product of a close interaction between orbital fibroblasts and T-cell lymphocytes. Various classes of immunomodulators (e.g. HLA antigens, CTLA-4, and cytokines) mediate this interaction. Polymorphisms in immunomodulator genes can alter the interaction between T-cells and orbital fibroblasts and impact disease susceptibility and progression.

**Table 1.** Summary of the Studies on Immunogenetics of Graves’ Ophthalmopathy

Polymorphism	Population	Sample Size	Study

**Cytokines**			

IL-3, rs40401	Chinese	GO (+) = 190	Zhu *et al*. [65], 2010
		GO (-) = 561	

IL-4, rs2070874	Chinese	GO (+) = 190	Zhu *et al*. [65], 2010
		GO (-) = 561	

IL-5, rs2069812	Chinese	GO (+) = 190	Zhu *et al*. [65], 2010
		GO (-) = 561	

IL-1β, rs3917368	Taiwan Chinese	GO (+) = 200	Liu *et al*. [81], 2010
		GO (-) = 271	

IL-1β, rs1143643	Taiwan Chinese	GO (+) = 200	Liu *et al*. [81], 2010
		GO (-) = 271	

IL-1α, rs1800587	Han Chinese	GO (+) = 190	Liu *et al*. [66], 2010
		GO (-) = 570	

IL-1β, rs16944	Han Chinese	GO (+) = 190	Liu *et al*. [66], 2010
		GO (-) = 570	

IL-4, -1098 T/G	Iranian	GO (+) = 50	Khalilzadeh *et al*. [62], 2010
		GO (-) = 57	

IL-4, -33 C/T	Iranian	GO (+) = 50	Khalilzadeh *et al*. [62], 2010
		GO (-) = 57	

TGF-β, +869 T/C	Iranian	GO (+) = 50	Khalilzadeh *et al*. [62], 2010
		GO (-) = 57	

TGF-β, +915 G/C	Iranian	GO (+) = 50	Khalilzadeh *et al*. [62], 2010
		GO (-) = 57	

TGF-β, +915 G/C	Iranian	GO (+) = 50	Khalilzadeh *et al*. [62], 2010
		GO (-) = 57	

IL-1α, -889 C/T	Iranian	GO (+) = 50	Khalilzadeh *et al*. [61], 2009
		GO (-) = 57	

IL-1 receptor antagonist	Iranian	GO (+) = 50	Khalilzadeh *et al*. [61], 2009
Mspa-1 11100 T/C		GO (-) = 57	

TNF-α, -238, G/A	Iranian	GO (+) = 50	Anvari *et al*. [63], 2009
		GO (-) = 57	

IFN-γ	Iranian	GO (+) = 50	Anvari *et al*. [63], 2009
UTR 5644, A/T		GO (-) = 57	

IL-8, rs2227306	Chinese	GO (+) = 164	Gu *et al*. [126], 2009
		GO (-) = 478	

IL-23R, rs2201841	North American Caucasian	GO (+) = 103	Huber *et al*. [127], 2008
		GO (-) = 111	

IL-23R, rs10889677	North American Caucasian	GO (+) = 103	Huber *et al*. [127], 2008
		GO (-) = 111	

IL-16	Chinese	GO (+) = 136	Gu *et al*. [128], 2008
(rs4778889-rs1131445-rs4778641)		GO (-) = 122	

TNF-α, -863C/A	Chinese	GO (+) = 54	Yan *et al*. [128], 2005
		GO (-) = 122	

TNF-α, -238G/A	Polish	GO (+) = 106	Bednarczuk *et al*. [70], 2004
		GO (-) = 122	

TNF-α, -1031 T/C	Japanese	GO (+) = 62	Kamizono *et al*. [68], 2000
		GO (-) = 111	

TNF-α, -863C/A	Japanese	GO (+) = 62	Kamizono *et al*. [68], 2000
		GO (-) = 111	

**CTLA-4**			

CTLA-4, +49 GA	Iranian	GO (+) = 105	Esteghamati *et al*. [85], 2009
		GO (-) = 100	

CTLA-4, -318 CT	Chinese	GO (+) = 33	Zhang *et al*. [129], 2006
		GO (-) = 56	

CTLA-4, -318 CT	Chinese	GO (+) = 142	Han *et al*. [130], 2006
		GO (-) = 121	

CTLA-4, +49 GA	Polish	GO (+) = 50	Frydecka *et al*. [86], 2004
		GO (-) = 49	

CTLA-4, +49 GA	British	GO (+) = 94	Vaidya *et al*. [87], 2003
		GO (-) = 94	

CTLA-4, +49 GA	British	GO (+) = 94	Vaidya *et al*. [87], 2003
		GO (-) = 94	

CTLA-4, +1822 CT	British	GO (+) = 94	Vaidya *et al*. [87], 2003
		GO (-) = 94	

CTLA-4, +49 GA	British	GO (+) = 129	Vaidya *et al*. [88], 1999
		GO (-) = 172	

**HLA**			

HLA-DRB1*120101	Korean	GO (+) = 17	Jang *et al*. [107], 2010
		GO (-) = 113	

HLA-DRB1*03 (DR3)	Italian	GO (+) = 54	Buzzetti *et al*. [92], 1999
		GO (-) = 38	

HLA-DRB1*07 (DR7)	German	GO (+) = 96	Badenhoop *et al*. [104], 1992
		GO (-) = 38	

HLA-DRB1*07 (DR7)	German	GO (+) = 96	Badenhoop *et al*. [104], 1992
		GO (-) = 38	

HLA-DRB3*01 (DR52)	German	GO (+) = 214	Boehm *et al*. [110], 1992
		GO (-) = 90	

HLA-DPB1*0201 (DPB2·1)	British	GO (+) = 43	Weetman *et al*. [99], 1990
		GO (-) = 48	

HLA-DRB1*04 (DR4)	Canadian	GO (+) = 64	Frecker *et al*. [105], 1988
		GO (-) = 69	

HLA-DRB1*03 (DR3)	Hungarian	GO (+) = 62	Frecker *et al*. [102], 1986
		GO (-) = 55	

HLA-B*08	Hungarian	GO (+) = 62	Frecker *et al*. [102], 1986
		GO (-) = 55	

HLA-B*08	Hungarian	GO (+) = 84	Stenszky *et al*. [101], 1983
		GO (-) = 79	

HLA-DRB1*03 (DR3)	German	GO (+) = 101	Schleusener *et al*. [131], 1983
		GO (-) = 49	

**Other Markers**			

CD103 , rs11652878	Chinese	GO (+) = 203	Liu *et al*. [119], 2010
		GO (-) = 281	

CD86, rs9831894	Taiwan Chinese	GO (+) = 200	Liao *et al*. [118], 2010
		GO (-) = 271	

Toll-like receptor-9, rs287084	Taiwan Chinese	GO (+) = 200	Liao *et al*. [117], 2010
		GO (-) = 271	

Toll-like receptor-9, rs352140	Taiwan Chinese	GO (+) = 200	Liao *et al*. [117], 2010
		GO (-) = 271	

Glucocorticoid receptor BcII allele	Hungarian	Severe GO = 70	Boyle *et al*. [116], 2008
		Mild GO = 25	

Protein tyrosine phosphatases, Non- Receptor Type 12	British	mild/moderate GO = 354	Syed *et al*. [114], 2007
rs1468682, rs4729535 rs17467232		GO (-) = 366	

NFκ, -94ins/del ATTG	Japanese	GO (+) = 123	Kurylowicz *et al*. [115], 2007
		GO (-) = 301	

ICAM-1, 1405A/G	Polish	GO (+) = 108	Kretowski *et al*. [113], 2003
		GO (-) = 127	
